# Evaluating the Use of Generative AI Videos for Health Self-Management of Older Adults: Mixed Methods Study

**DOI:** 10.2196/88005

**Published:** 2026-03-04

**Authors:** Ting Liu, Patrick Pang, Yiming Taclis Luo, Dana McKay, George Buchanan, Shanton Chang

**Affiliations:** 1 Faculty of Applied Sciences Macao Polytechnic University Macao China; 2 School of Computing Technologies Royal Melbourne Institute of Technology University Melbourne Australia; 3 School of Computing and Information Systems The University of Melbourne Melbourne Australia

**Keywords:** generative AI, GenAI videos, older adults, health self-management, mobile phone

## Abstract

**Background:**

Aging is a pressing global issue, and older adults need to build up their knowledge to manage their health. Insufficient self-efficacy and low acceptance of technology hinder their ability to use emerging technologies for self-management.

**Objective:**

This study explored the potential of generative artificial intelligence (GenAI) videos in the health management of older adults.

**Methods:**

We developed a video-based GenAI prototype, AIHealthV. This study used a mixed methods approach, enrolling 20 older adult participants (aged 60 to 80 years) in 3 rounds of iterative workshops. Data collection included pre- and postquestionnaires, in-depth interviews, prompt text records, and the generated video content. Qualitative data were analyzed using the 6-stage reflexivity thematic analysis method by Braun and Clarke, and quantitative data were analyzed using the Wilcoxon signed-rank test.

**Results:**

The findings revealed that GenAI videos can enhance older adults’ self-efficacy and technology acceptance, reduce cognitive load, and simultaneously meet their health management needs. The multimodal content generated by GenAI makes health information more comprehensible and thus improves the accessibility of health knowledge. Following the workshop, the interaction between older adults and AIHealthV exhibits a trend of exploration, adaptation, and verification.

**Conclusions:**

Despite ethical, privacy, and usability concerns, AIHealthV has been proven to be beneficial in improving the health management capabilities of older adults. This paper also provides practical insights for developing artificial intelligence health tools tailored to older adults as GenAI tools continue to prevail.

## Introduction

With the acceleration of global aging, the World Health Organization projects that the worldwide population aged 65 years and older will increase to 1.5 billion by 2050 [1]. Physiological declines associated with aging, including deteriorating physical functions, sensory impairments, and memory loss, alongside this increase, have increased older adults’ needs for self-health management, particularly in disease prevention, health monitoring, and chronic disease management [1]. Technology offers opportunities to support self-management; however, research has found that older adults encounter significant difficulties in accessing printed or online health information, particularly in technologically complex and information-overloaded environments [2]. Technology-enabled precision health information, for example, on-demand generation of health educational content, can improve older adults’ health management and decision-making abilities [3]. The rapid development of artificial intelligence (AI) sheds light on the generation of tailor-made information for older adults’ self-health management.

Older adults are gradually emerging as a significant user group of digital health technologies, which enable them to access health-related information and resources on time, thereby facilitating better health management and chronic disease control [4]. Accessing health knowledge through digital technologies not only enhances older adults’ health management capabilities but also strengthens their sense of technological control [5]. However, their persistent reliance on traditional health knowledge acquisition channels (eg, printed materials, doctor visits, or face-to-face classes) also limits the potential of digital technologies in this domain [6]. Studies indicate that some seniors use AI voice assistants (eg, Amazon Echo and Google Home) to access health information [7]. In contrast, others rely on AI conversational agents and robotic systems for health-related queries [8]. Despite their potential, text- or image-based health AI applications often present interaction challenges for older users due to limited comprehension abilities or complex interfaces [9]. Complicated operations and navigation structures further hinder adoption [2]. Additionally, concerns about data privacy and security are prevalent among older adults when using AI [10], with technological distrust potentially reducing acceptance [11].

The capabilities of generative artificial intelligence (GenAI) to create on-demand informational material in a variety of formats, including text, video, or images, offer new opportunities for tailored digital education [12]. The emergence of GenAI has opened new avenues for efficient health knowledge dissemination and precision personalized education [13]. Furthermore, GenAI’s capacity for dynamic content modification and optimization enhances the quality and user adaptability of health knowledge outputs through iterative refinement [14]. AI-generated health videos offer high flexibility and adaptability, enabling the creation of personalized video content based on user backgrounds, such as educational level and health needs, such as disease prevention or treatment decision-making [15].

GenAI is revolutionizing the creation and dissemination of health information, particularly in producing high-quality, dynamic content. Personalized health videos generated by GenAI have been preliminarily applied in patient education and health promotion, such as disease-specific tutorials [16]. GenAI technologies can dynamically generate personalized, easily comprehensible health information, particularly through video, a visually rich medium that compensates for the interpretive challenges of text-based content [17]. These videos dynamically tailor information to user needs, outperforming static content in engagement. Videos as a form of educational materials can improve adults’ self-efficacy and reflect their critical thinking skills [18], but this is not necessarily true for older adults. Beyond dynamic generation, GenAI’s capacity for scenario-specific personalization enhances its utility in health education [19]. Furthermore, generative models integrated with sentiment analysis (eg, detecting user satisfaction or confusion) can swiftly identify older adults’ preferences for video design and functionality, optimizing information delivery [20]. GenAI’s capacity for dynamic content modification and optimization enhances the quality and user adaptability of health knowledge outputs through iterative refinement [14]. By combining GenAI with user-driven optimization, health communication strategies for older adults can be refined more effectively [21].

While technology offers many opportunities, declines in information processing speed, sensory functions, and memory capacity create numerous barriers for older adults in accessing and comprehending health information via digital technologies [2]. Additionally, research reveals that older adults exhibit deeper fears regarding data security and potential privacy breaches, which further hinder their access to and use of emerging internet-based digital health management tools [22]. Technophobia and heightened concerns about usability and privacy issues represent significant barriers to the adoption of digital health among older adults [12]. These factors contribute to the challenges of promoting the use of AI technologies in general.

Self-efficacy plays a fundamental role in older adults’ use of digital health technologies. According to the social cognitive theory by Bandura [23], an individual’s belief in their ability to perform specific tasks influences their willingness to try and persist with new behaviors. In the context of technology adoption, Kim [24] found that older adults with higher technological self-efficacy showed greater persistence when facing initial usage difficulties. The development of technological self-efficacy in older adults follows unique patterns. Unlike younger users who typically acquire digital skills through independent exploration, older adults usually benefit more from organized, guided learning experiences [25]. Schroeder et al [26] emphasized that successful skill-building interventions for this population should include progressive challenge advancement, immediate feedback mechanisms, and opportunities for mastery experiences, which have been proven to enhance self-efficacy [27]. This self-efficacy then becomes a key determinant of technology acceptance. The perceived ease of use (PEOU) influences adoption decisions, especially for older adults who may view technological interfaces as complex [28]. Research by Malureanu et al [29] shows that enhanced self-efficacy directly improves perceived usefulness (PU) and ease of use, the 2 core elements of technology acceptance. This relationship creates a positive feedback loop: as older adults gain confidence through successful experiences, their willingness to adopt more advanced features increases [30]. The interaction between self-efficacy and acceptance has important practical implications. GenAI offers the potential for high self-efficacy, through simple interaction, for older adults, a potential that merits further exploration.

**Figure 1:**
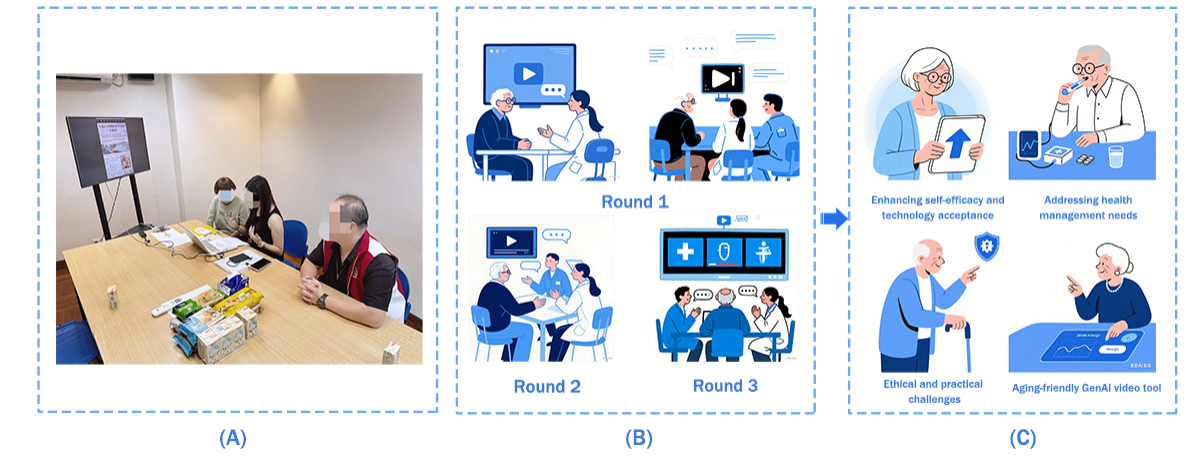
Summary of this paper. GenAI: generative artificial intelligence.

Static output modes in video generation tools neglect user feedback, failing to adapt interactively for maximal information retention. While this approach has theoretical benefits for older adults, these benefits warrant empirical analysis as in this paper. In this paper, we use “GenAI” to refer specifically to its use in creating health promotion videos. The usefulness of this application in supporting health education for older adults is currently underexplored. Figure 1 is a summary of this paper: Figure 1A shows that workshops were implemented through the following process to obtain requirements from the participants. Prompts and health videos were generated collaboratively between participants and researchers. Figure 1B shows that 3 rounds of videos were generated in a session. Videos were regenerated, discussed, and evaluated in each round. Figure 1C shows key research findings.

To systematically explore the potential of GenAI videos in supporting older adults’ health management, this study addresses three key research questions: (1) How can AI-generated videos create an impact on older adults’ health management? (2) What is the role of AI-generated videos in the health management processes of older adults? (3) What are the issues and concerns faced by older adults when using GenAI technologies?

## Methods

### Ethical Considerations

This study was approved by the Human Ethics Committee of Macao Polytechnic University (HEA006-FCA-2025). All procedures followed the guidelines of the principles of the World Medical Association Declaration of Helsinki. All participants provided written explicit informed consent before participation. All data were deidentified. Before the data collection began, the consent form was obtained through an offline paper-based method, and participants were informed about the voluntary nature of this study and their right to withdraw at any time without penalty or loss of benefits. All participant data and visual materials (including figures, video samples, and prototype interface screenshots) in this manuscript have been fully deidentified to eliminate any identifiable personal information, strictly adhering to privacy and confidentiality protection requirements. To ensure the accuracy and safety of generated content, community workers who worked at the community center where this study was conducted were invited to the workshops. They conducted manual reviews of GenAI-generated videos, focusing on verifying the accuracy of health information and mitigating the risk of misleading information. Specifically, these reviews occurred at two critical checkpoints: (1) immediately after the initial script and plot generation to flag potential medical inaccuracies, and (2) after the final video rendering to ensure visual-audio alignment and content safety. All videos required confirmation by community workers to ensure that they did not violate any scientific medical knowledge before they were presented to participants. Additionally, before the workshops, participants were explicitly informed that “AI-generated content should be used in conjunction with professional advice,” and the consent form emphasized the limitations of GenAI and the potential of inaccurate information. Participants voluntarily signed up for the workshops and chose a suitable timeslot. They did not receive any additional compensation, but snacks and drinks were provided in the workshop.

### Prototype Development

We developed a prototype of a GenAI video tool tailored to older adults, named AIHealthV. This prototype allowed us to customize GenAI for better meeting older adults’ needs for health management. For example, AIHealthV integrated voice input and keyword suggestions to reduce the barrier for content creation, as many older adults were reluctant to type; video outputs supported adjustments, including playback speed and voice selection, as well as subtitle fine-tuning with adjustable font sizes. These usability considerations were considered crucial for carrying out our study successfully with the older adult cohort.

For the technical implementation of this prototype, we integrated different software libraries as they have separate functions. The video generation application was built using Python (version 3.12; Python Software Foundation; for the backend) and React (version 18.2; Meta Platforms, Inc; for the frontend), which interconnects with SiliconFlow AI services for image and video generation. The default video length was set to 3-5 minutes, with output parameters optimized for high-contrast mobile viewing to suit the visual needs of older users. The prototype also featured a prompt generator embedded with preloaded health templates (eg, hypertension management and sleep improvement), which leveraged the GPT-4o (OpenAI) model for script generation.

The prompt template followed the structure with content of the role, background, health issue, and constraint, for example, “I am 70 years old with diabetes and want to learn about a sugar-controlled healthy diet, focusing on [specific constraint].” In this way, the video plots and transcripts generated by the prototype were more health-related and accurate. Furthermore, to make the prototype more trustworthy and encourage older adults to share their health concerns, we used a friendly, human-like voice for feedback with a simplified and conversational interface instead of a technical one. These human-centric design elements help reduce the anxiety that older adults might feel when using the new technology, making them more willing to “talk” to the AI and disclose their real health needs [31].

**Figure 2:**
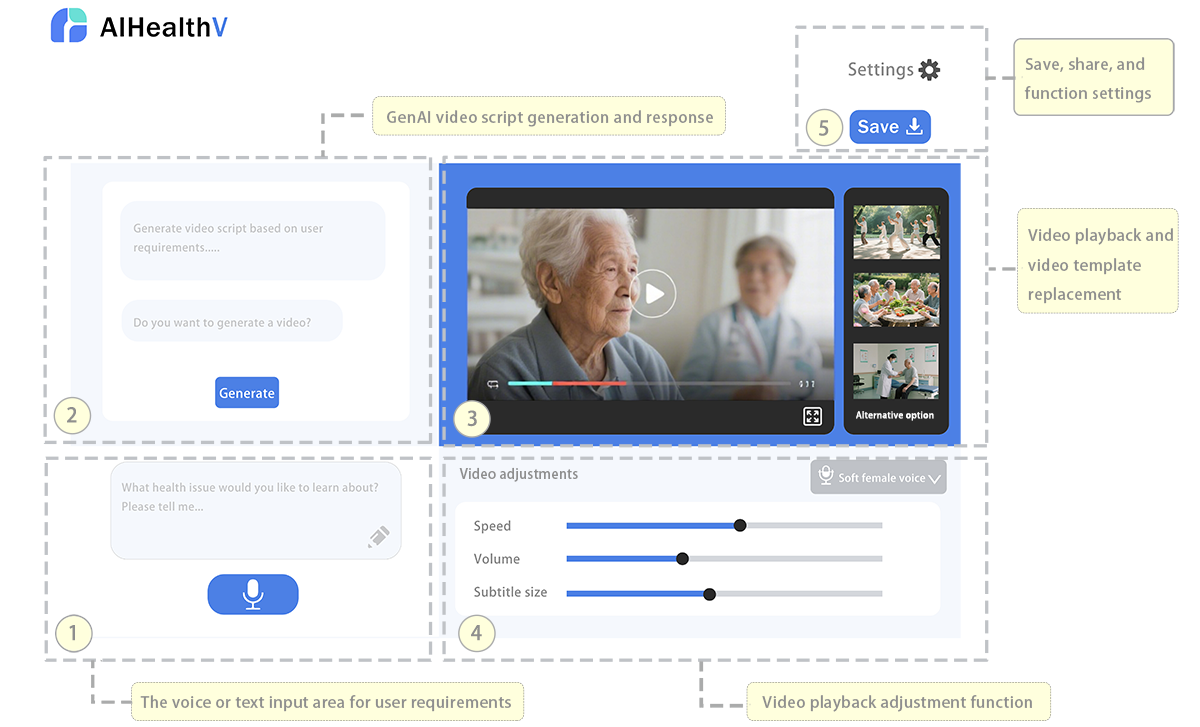
The prototype for generating health-related videos with GenAI. GenAI: generative artificial intelligence.

The user interface design adopts a step-by-step guidance process: (1) a multimodal interactive input area supporting voice and text input, which is used to generate video plots; (2) the output of video plots which are used for video generations, and the content can be modified for fine-tuning video output; (3) a video preview area including multiple alternative videos; (4) features for adjusting elements such as voice, speed, volume, and subtitle size; and (5) generic functions such as settings, saving, and sharing. Figure 2 demonstrates the design of AIHealthV.

### Participant Recruitment

With the assistance of community partners, we recruited 24 older adults aged 60 years and older from nearby communities through offline posters to participate in an iterative workshop. The inclusion criteria used were that participants were 60 years of age or older and had the basic operational ability to use smart devices such as smartphones and tablets. Of the 24 recruited participants, 4 were excluded from the final analysis: 3 withdrew before the pretest due to sudden scheduling conflicts, and 1 failed to complete the full iterative workshop rounds due to physical fatigue. Ultimately, results from 20 participants were included in this study. The purpose of this sample selection is to consciously focus on different population characteristics, such as the different levels of education, living conditions, and health needs (such as high blood pressure, joint health, and sleep disorder) of participants, to maximize the reliability of the information. All participants expressed motivation and a need to use GenAI and health videos for better self-health management. Participants had a range of health conditions (chronic disease, good health, and mild illness) and lived in a variety of residential situations (eg, living alone, with family, or in a nursing facility). In addition, community workers also participated in this study. They worked in community-based health, had a basic understanding of personal health management, and had experience assisting older adults with technology tools. It is the role of community workers to professionally review the content generated by GenAI to effectively circumvent the risk of AI generating erroneous or inaccurate medical information, thereby ensuring the safety and reliability of all discussed information.

### Procedure

**Figure 3:**
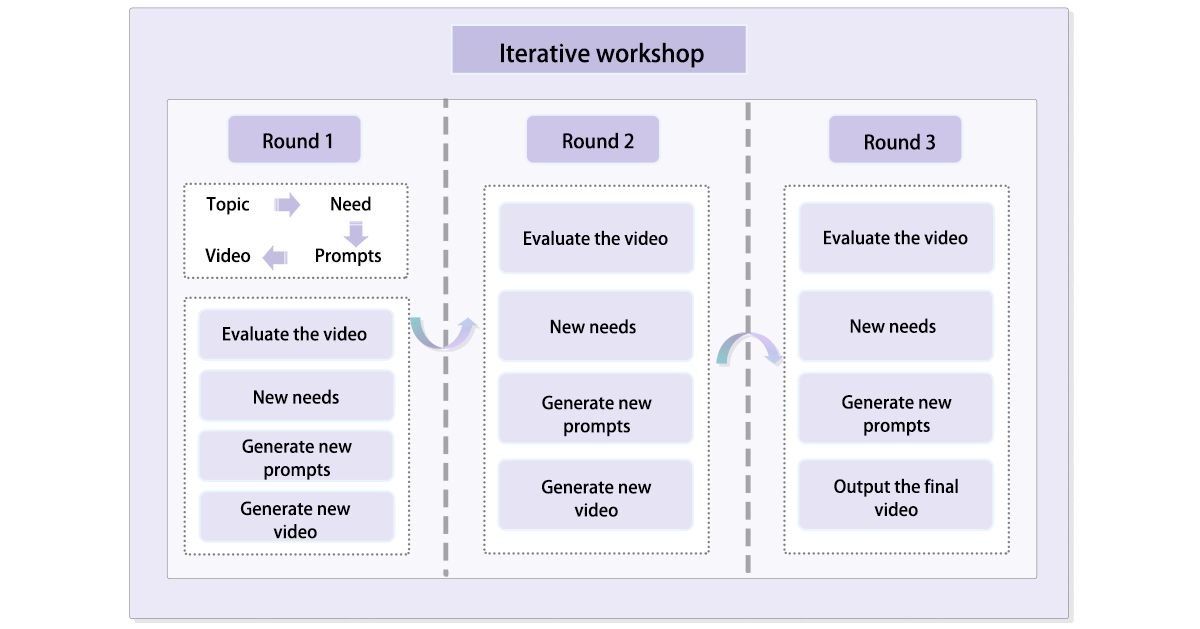
The procedure of workshop implementation.

Each workshop lasted for about an hour for a single participant and consisted of three phases: (1) pretest, (2) iterative workshop, and (3) posttest. The pretest phase (about 20 minutes) used questionnaires and interviews to collect participants’ subjective evaluations regarding health knowledge needs, technological proficiency, and self-efficacy. In addition, each participant received a 15-minute introduction to the fundamental principles of GenAI and our prototype for video generation. Then, the iterative workshop was carried out with the following procedure (Figure 3). First, participants identified health topics based on personal interests and articulated their requirements across 5 video dimensions (visual, auditory, narrative, cognitive, and emotional) [32], which were then translated into prompts for video generation by the researcher to produce the first video. Second, after each video generation, community workers reviewed the content and flagged potential issues in the video (eg, incorrect medical advice or misleading claims) if there were any. This step was put in place to ensure discussions were grounded in reliable information. Finally, participants then reviewed the initial video generated by AI, discussed their experiences, provided feedback, and proposed new requirements, which were refined into a revised version of prompts for the generation of the second video.

This iterative process was repeated for a total of 3 cycles, resulting in a final video incorporating the feedback from all rounds.

The posttest phase (about 20 minutes) involved another series of questionnaires and interviews to evaluate participants’ satisfaction with the final video, as well as the changes in self-efficacy and technology acceptance. The entire research process emphasized iterative improvements to the generated videos to ensure progressive alignment between participant needs and the capabilities of the GenAI technology.

### Data Collection and Analysis

This study adopts a mixed methods approach, integrating both qualitative and quantitative data for analysis. Data were primarily collected from two sources: (1) pretest and posttest questionnaires, along with interview transcripts from the iterative workshops, and (2) prompts and generated videos recorded in the system. To mitigate potential social-desirability effects stemming from the workshop setting, interviews were conducted by researchers in a neutral community environment, with a strong emphasis to participants that their negative feedback was essential for system improvement.

Interview transcripts were analyzed using the 6-phase reflexive thematic analysis (RTA) by Braun and Clarke [33]. RTA was specifically chosen for this qualitative inquiry due to its emphasis on the researcher’s subjective and reflexive involvement in meaning-making, allowing us to generate rich, interpretative themes beyond simple data summation, focusing on “what is said and why” in the context of emerging GenAI technology. The RTA process involved: (1) familiarization (transcribing all interviews and reading the data for initial immersion); (2) generating initial codes (identifying semantic and latent features across the dataset); (3) searching for themes (grouping codes into meaningful broader categories); (4) reviewing themes (checking themes against the coded extracts and the entire dataset to ensure internal consistency and external heterogeneity); (5) defining and naming themes (creating clear, concise, and academically rigorous themes, as presented in Table 1); and (6) producing the report. The coding process was performed collaboratively by two researchers to ensure analytical rigor through constant comparison and to minimize individual bias.

**Table 1:** Theoretical dimensions and thematic coding.

Theoretical dimension and theme		Sample quotations
**Self-efficacy**		
	Gaining confidence using GenAI^a^ videos	“I believe I can complete the operation with guidance.” (E1) “I am capable of learning as long as someone teaches me.” (E6)
	Instructions needed for using GenAI videos	“I need step-by-step instructions to use it.” (E4) “I need specialized guidance on using GenAI.” (E7)
	Ease of use	“This video generation process feels simpler than other mobile apps I use.” (E3) “The operation feels as simple as using WeChat.” (E2)
**Technology acceptance**		
	Perceived usefulness	“The generated video effectively explains hypertension symptoms, which is very useful for me.” (E1) “The health videos help address my medical concerns.” (E5)
	User-friendliness	“The interface is more user-friendly than expected.”(E8)
	Intention to use	“I hold an open attitude toward learning GenAI and creating videos, and I’m willing to try.” (E3) “Gradual adoption is needed.” (E9)
	Perceived risks	“I worry about privacy breaches when using GenAI.” (E15) “GenAI outputs require human review.” (E19)
**Health management**		
	Ways of understanding information	“Dynamic visuals are more intuitive than static images or text.” (E1) “Animated video explanations are clearer than doctors’ charts.” (E3)
	Providing personalized information	“It can provide personalized dietary recommendations.” (E4) “GenAI gives exercise guidance tailored to my current physical condition.” (E7)
	Needs of management plans	“I hope GenAI can help me develop fitness plans.” (E1) “I want GenAI to provide sleep improvement plans.” (E12)
	Requiring further medical or professional advice	“I’ll consult doctors before adopting GenAI suggestions.” (E5) “Family or professionals should review certain content.” (E9)

^a^GenAI: generative artificial intelligence.

The coding process was performed collaboratively by 2 researchers who independently coded the data before holding coordination meetings to refine reflexive interpretations and ensure coding consistency. This methodical approach adheres to the standards for reporting qualitative research guidelines. The research team was external to the community center, maintaining a critical distance to minimize potential bias. Thematic saturation was determined when no new codes or subthemes emerged during the analysis of the final 3 participant interviews. We analyzed interview transcripts from all 20 participants, reaching thematic saturation where no new themes related to self-efficacy, acceptance, or concerns emerged in the final interviews. Thematic saturation was determined when no new codes or subthemes emerged during the analysis of the final 3 interviews.

Prompts and videos were analyzed using an inductive content analysis [34] to categorize the evolution of user requests and the content sophistication across the 3 rounds. In addition, comparative analysis was conducted to assess the iterative evolution of prompts and videos.

For quantitative analysis, the pretest and posttest questionnaires were designed to be relatively short and included only a few dimensions, so that older adults could complete them without difficulties. As older adults’ needs and preferences for using technologies can be unique, we chose usability dimensions that focus on aging [26]. For this reason, we referred to the literature studying older adults’ acceptance of technologies in health care to select appropriate dimensions for our questionnaire. As suggested by recent research and meta-analysis [24,28], we included the following constructs in our study: PU (3 items) refers to the perceived improvement in health management; PEOU (3 items) assesses the effort required; and behavioral intention (BI, 2 items) measures the future usage likelihood. In addition, to gauge the effectiveness and efficacy of older adults in adopting GenAI for health management, we included additional measurements of technology confidence (TechConf, 4 items) [35], task mastery (TaskMastery, 2 items) [25], and the learning of health information (HealthLearn, 3 items) [36]. Internal consistency for these scales was verified, with all constructs demonstrating acceptable reliability (Cronbach α>0.70). The questions were presented to the participants in the pre- and postphases, then Wilcoxon signed-rank tests were used to examine significant differences between pretest and posttest questionnaire results. To evaluate effect sizes, we computed Cohen *d* and additionally reported the Wilcoxon-compatible effect size *r* (calculated as Z divided by the square root of the sample size). In this study, items within core dimensions showed substantial effect sizes; for instance, Cohen *d* values for PEOU items ranged from 0.96 to 1.53 (*r*=0.75-0.86), and the BI items reached a Cohen *d* of 1.05 (*r*=0.81). The full list of questions can be found in Multimedia Appendix 1.

### Sample

We recruited 20 older adults residing in nearby communities to participate in iterative workshops of GenAI videos. The participants were aged 60-84 (mean 70.15, SD 6.90) years, possessed basic skills of using smartphones, but had no experience with GenAI. This inclusion criterion ensures that their initial attitudes and user acceptance toward GenAI were not influenced by previous exposure. To enhance the heterogeneity of our sample, the participants reported diverse health statuses (eg, healthy, with chronic or mild conditions), living arrangements (eg, living alone or with family), and educational background (from primary school to university). They were also interested in a wide range of health topics, such as the 3 hyper-conditions, cardiovascular health, sleep disorders, and exercise-related topics. Table 2 lists the demographic information of the participants.

**Table 2:** Demographic information of our sample.

ID	Age (years)	Gender	Self-reported health status	Living arrangement	Education level	Health topics of concern
E1	60	Woman	Healthy	Alone	High school	Hypertension, hyperlipidemia, and hyperglycemia
E2	80	Woman	Chronic condition	Alone	Primary school	Sleep disorders
E3	78	Woman	Healthy	Living with family	Junior high school	Cardiovascular health
E4	77	Men	Chronic Condition	Living with family	High school	Hypertension, hyperlipidemia, and hyperglycemia
E5	72	Woman	Chronic condition	Living with family	Primary school	Healthy diet
E6	61	Woman	Chronic condition	Living with family	University	Physical wellness
E7	69	Woman	Chronic condition	Living with family	Junior high school	Obesity
E8	73	Woman	Chronic condition	Alone	Primary school	Healthy diet
E9	67	Woman	Healthy	Living with family	High school	Joint health
E10	76	Woman	Healthy	Alone	Primary school	Joint health
E11	74	Men	Mild condition	Living with family	Junior high school	Exercises
E12	69	Men	Chronic condition	Alone	Junior high school	Healthy diet
E13	73	Woman	Chronic condition	Living with family	Primary school	Joint health
E14	62	Woman	Mild condition	Living with family	University	Thyroid issues
E15	84	Woman	Chronic condition	Alone	College diploma	Sleep disorders
E16	67	Woman	Healthy	Alone	Junior high school	Joint health
E17	62	Woman	Healthy	Lives with family	Primary school	Hypertension, hyperlipidemia, and hyperglycemia
E18	72	Woman	Healthy	Lives with family	High school	Healthy diet
E19	62	Woman	Healthy	Alone	Primary school	Joint health
E20	65	Men	Chronic disease	Alone	University	Sleep disorders

## Results

### Quantitative Impact on Confidence and Learning

Through Wilcoxon signed-rank tests, we analyzed the impact of workshops on multiple dimensions among older adults. The results demonstrated consistently positive mean differences across all dimensions (ranging from 0.375, SD 0.582, to 0.917, SD 0.571) after the workshops, with all reaching statistical significance (*P* ≤.01). Notably, PEOU, PU, BI, technical confidence (TechConf), and the influence on health information learning (HealthLearn) exhibited substantial mean increases. Table 3 shows the results of the Wilcoxon signed-rank tests.

**Table 3:** Summary of Wilcoxon signed-rank test results.

Item	Mean difference (SD)	Wilcoxon statistic	*P* value
PU^a^	0.867 (0.652)	0	.001^b^
PEOU^c^	0.917 (0.571)	0	.001^b^
BI^d^	0.575 (0.712)	12	.003^b^
TechConf^e^	0.65 (0.455)	0	.001^b^
TaskMastery^f^	0.375 (0.582)	13.5	.01^b^
HealthLearn^g^	0.717 (0.544)	0	.001^b^

^a^PU: perceived usefulness.

^b^Statistically significant.

^c^PEOU: perceived ease of use.

^d^BI: behavioral intention.

^e^TechConf: technical confidence.

^f^TaskMastery: task mastery.

^g^HealthLearn: health information learning.

To evaluate the practical significance of these changes, Cohen *d* was calculated. The results showed large effect sizes for PEOU (*d*=1.61), PU (*d*=1.33), and the influence of health information learning (*d*=1.32). Medium to large effects were also found in technical confidence (*d*=1.43), BI (*d*=0.81), and task mastery (*d*=0.64). These results indicated that these improvements were not only statistically significant but also practically meaningful. Table 4 lists the results of each item and its Cohen *d* tests.

**Table 4:** Wilcoxon signed-rank test and Cohen *d*.

Item	Mean difference (SD)	Wilcoxon statistic	*P* value	Cohen *d*
PU1^a^	0.75 (0.79)	0	.002^b^	0.95
PU2	1 (1.03)	0	.002^b^	0.97
PU3	0.85 (0.81)	0	.002^b^	1.05
PEOU1^c^	1.1 (0.72)	0	.001^b^	1.53
PEOU2	0.85 (0.81)	0	.002^b^	1.05
PEOU3	0.8 (0.83)	7	.001^b^	0.96
BI1^d^	0.3 (0.92)	37.5	.16	0.32
BI2	0.85 (0.81)	0	.001^b^	1.05
TechConf1^e^	0.5 (0.51)	0	.002^b^	0.97
TechConf2	0.75 (0.79)	0	.002^b^	0.95
TechConf3	0.7 (0.86)	0	.004^b^	0.81
TechConf4	0.65 (0.75)	0	.004^b^	0.87
TaskMastery1^f^	0.35 (0.75)	10	.05	0.47
TaskMastery2	0.4 (0.75)	11	.03^b^	0.53
HealthLearn1^g^	0.8 (1.06)	4.5	.005^b^	0.76
HealthLearn2	0.6 (0.6)	0	.001^b^	1
HealthLearn3	0.75 (0.72)	0	.001^b^	1.05

^a^PU: perceived usefulness.

^b^Statistically significant.

^c^PEOU: perceived ease of use.

^d^BI: behavioral intention.

^e^TechConf: technical confidence.

^f^TaskMastery: task mastery.

^g^HealthLearn: health information learning.

### Older Adults’ Experiences of Technology

#### Overview

We qualitatively analyzed the interview transcripts of 20 participants as data saturation was reached, and identified themes around self-efficacy, technology acceptance, and health information comprehension. Data analysis followed the 6-phase method for RTA by Braun and Clarke [33]. RTA emphasizes the researcher’s reflexive involvement in the data analysis process, where subjective interpretation is central to understanding and generating meaning from qualitative data [37]. In terms of the changes in self-efficacy, we found that older adults gained confidence using GenAI from the workshops. Meanwhile, they expressed the need for more instructions on using GenAI. The ease of use of the GenAI video technology under simple and clear instructions also contributed to the improvement in self-efficacy.

Another category of emerging themes was related to technology acceptance. Participants perceived that AI-generated videos were useful, and the GenAI tool was user-friendly. The workshops demonstrated the applications of GenAI videos in health management, and participants tended to use them in the future after witnessing the positive side of GenAI. At the same time, they pointed out the potential risks of such AI tools.

From a health management perspective, older adults found that GenAI videos changed their ways of understanding health information and allowed them to retrieve personalized information. However, the AI tool warranted more improvements, such as providing health management plans and integrating validation or reviews from medical and professional experts to ensure the accuracy and reliability of content. Table 1 summarizes the theoretical dimensions, themes, and sample quotations to support the findings. We use the following subsections to offer further insight into each theme.

#### Improvements in Self-Efficacy

Through content analysis of interview transcripts, it was found that the self-efficacy of participants exhibited a significant upward trend after engaging with the GenAI video tool. Before the workshops, 12 (60%) participants explicitly expressed concerns about the difficulties of GenAI technologies because of their ages. Many participants mentioned concerns such as, “my memory has deteriorated, and I can’t learn new things as quickly as before” (E4), and “I need my children’s guidance to operate these new technologies” (E8). This low confidence stemmed from doubts about their digital capabilities and fear of complicated user interfaces. After the workshop, 14 (70%) participants reported that the iterative workshops helped them to use GenAI. For instance, participants noted, “by following your instructions and prompts step by step, I was able to complete the task” (E3) and “I could better understand the content [after the workshop]” (E12).

In the initial workshop stage, many participants demonstrated a strong reliance on external support, which meant that they hoped to complete learning through vocal guidance or assistance from other people. After the workshop, 12 (60%) participants reported that they could independently use our GenAI tool to generate videos or adjust various settings. As 1 participant stated, “[other people’s] guidance when making mistakes enables success” (E5).

Before the workshops, 8 (40%) participants perceived GenAI video generation as a highly challenging task. The workshop helped to mitigate cognitive load through template-based prompt designs (eg, preset health-related prompting templates) and video revisions with feedback iterations. The postworkshop interviews showed that 16 (80%) participants expressed the ease of use of GenAI tasks. For example, “after the entire process, I [have] found [that] GenAI video generation simpler than I originally imagined” (E11) and “I now believe I can attempt creating my health videos” (E14).

#### Increase in Technology Acceptance

After the workshops, 18 (90%) participants emphasized the PU of the generated content was useful for them to manage their health, for example, “the GenAI-generated health video comprehensively explains hypertension knowledge and the content is practical” (E1) and “my legs are weak and I don’t know what exercises suit me. GenAI recommended exercises tailored to my condition” (E5).

Many participants commented on the user-friendliness of GenAI features. Among them, 16 (80%) participants mentioned the advantages of adaptable user interfaces (such as adjustable subtitle sizes) and the obvious design for specific functions (such as dedicated buttons for AI interaction). Other participants compared the GenAI with other commonly used mobile phone apps, for instance, “the operations are simple and similar to WeChat” (E2), which further contributed to the intention of using the technology.

While sentiment was generally positive, 5 (25%) participants expressed privacy concerns, including the possible leaks of personal data and the incorrect information provided by GenAI. For example, a participant said, “I worry about privacy breaches when using GenAI” (E15), while others suggested, “GenAI outputs require human review” (E19) or “professionals should review certain content” (E9).

#### Meeting Health Management Needs

GenAI videos were reported to help older adults better understand health information. A total of 15 (75%) participants described that AI-generated videos were a better way to present health knowledge, for example, “videos explain health knowledge better” (E3), and 11 (55%) participants mentioned that self-caring demonstrations generated by the GenAI were better, for example, “scenario-based demos in videos are more lively than [the demos from] medical experts” (E11).

Furthermore, at least 12 (60%) participants mentioned that GenAI video tools could provide personalized information, for instance, E12 reflected that the AI-generated video “taught how to do daily calorie calculations [for myself],” while many others mentioned that the GenAI could offer information about “healthy diets” and “hypertension control.”

Despite the positive feedback, participants highlighted the need for the GenAI to generate health management plans for older adults saying, “I hope GenAI can help me develop fitness plans” (E1), and “I want GenAI to provide sleep improvement plans” (E12). In addition, some participants suggested that they would not rely solely on the information provided by the GenAI. Instead, they will “consult doctors before adopting GenAI’s suggestions” (E5).

### Interaction Changes With Experience: Prompt Analysis

#### Overview

With the analysis of a total of 60 prompts collected in all workshops, the changes of prompts across 3 rounds of video generation demonstrated the progressive trajectory of older adult–AI interaction from exploration to decision-making support. The details in each round are shown below.

#### First Round

In the first round, older adults demonstrated an exploratory attitude toward AI-generated videos. Their understanding of GenAI began at an early stage, and their core concerns centered on exploring the basic capacities of the GenAI tool. Their prompts contained questions about privacy concerns and the accuracy of answers to explore the accuracy and reliability levels of the GenAI tool. Below are examples of this category of prompts:

I'm 77 years old. I have Hypertension, Hyperlipidemia, and Hyperglycemia. I want to know how diabetes can be prevented. Will my personal information be disclosed?

E4

I was 62. I lumped my thyroid. I want to know if there is a risk. Is the health video content [that] you provide correct?

E17

#### Second Round

In the second round, older adults focused on functional adaptation, and the prompts were changed accordingly. With a preliminary understanding of the technology, their focus shifted toward whether GenAI could precisely satisfy individual needs and whether the information was simple enough to understand. Prompts emphasizing personalized recommendations and requiring easy-to-understand information revealed their needs. The following are samples of the prompts:

Help me generate a video about joint health care. This video is a bit complicated. Please make it simpler and more understandable.

E16

I want to know how to lose weight healthily. Please recommend some diet and exercise videos that are suitable for my specific health condition.

E18

#### Third Round

In the final round, after asking the GenAI to provide personalization, older adults escalated to the level of asking for information to support decision-making. For instance, older adults asked for the content to support the discussion during consultations, or the content reviewed by human experts that could be shown to family members. The following shows the excerpts from such prompts:

I want to know about the issue of obesity among the elderly. Which method is better for losing weight, traditional Chinese medicine or Western medicine? … Are the suggestions you gave me the result of a review by relevant experts?

E7

I want to know about digestive problems in the elderly. My stomach often feels uncomfortable after eating. … Can dizziness only be caused by low blood sugar? Please help me make a detailed video about a healthy diet and lifestyle. Please provide some health suggestions that experts have reviewed.

E5

### Results of Generated Videos’ Analysis

**Figure 4:**
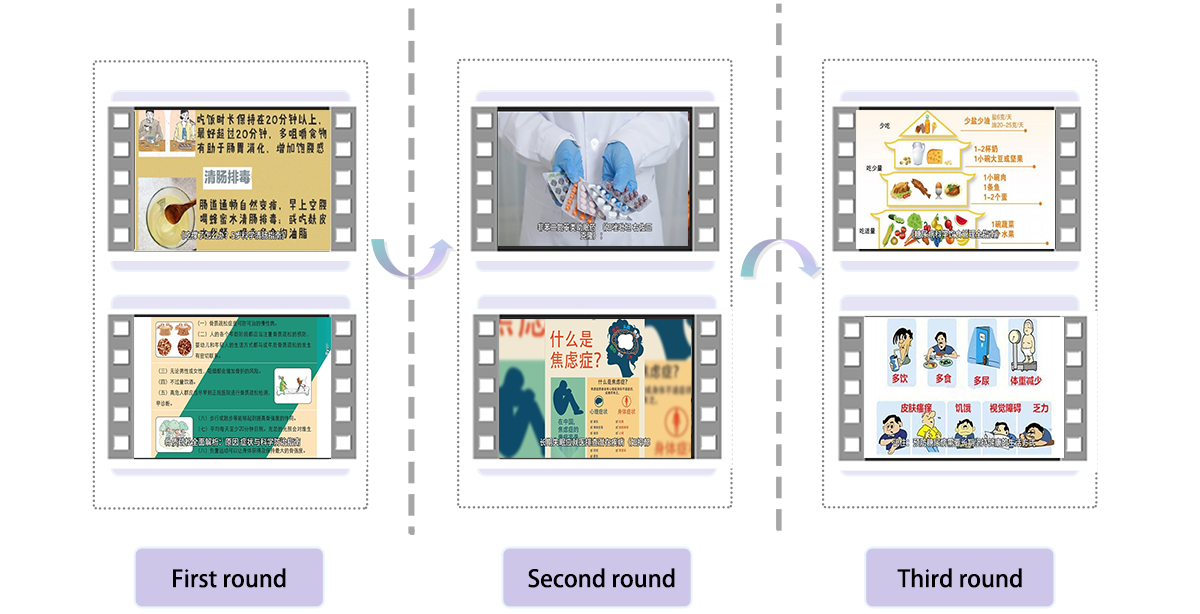
Sample videos in different rounds of the workshops.

The analysis of 60 videos produced across 3 rounds of workshops revealed the patterns evolving from vague to concrete. In the first round, participants engaged in exploratory attempts with ambiguous needs. At this stage, most videos consisted solely of text-based captions with static images. Then, the second round saw participants, who had gained preliminary experience with the GenAI tool, begin proposing more concrete demands. The video content at this stage started incorporating scenario-based plots, with a significant portion focusing on specific topics such as health management and medication reminders. However, the explanations about these topics remained vague. In the final iteration, the videos became more sophisticated, with most presenting comprehensive health solutions with the customization of target audiences, content formats, and risk management strategies. Figure 4 shows the video samples in 3 rounds of iterations. Chinese is displayed because the screenshots are captured from the original video clips.

## Discussion

### Principal Findings

This study systematically investigates the application and potential of GenAI video technologies and our prototype AIHealthV in older adults’ health management. The research captures the dynamic evolution of older adults’ interactions with GenAI and reveals how their needs progress from basic exploration to sophisticated decision-making support. Our findings provide novel insights into the role of AI-generated videos in reducing cognitive load and enhancing health information comprehension among older populations, while highlighting the importance of understanding their concerns in using GenAI technologies.

### Interaction and Multimodality: Cognitive Accessibility and Self-Efficacy

The research indicated an improvement in older adults’ self-efficacy and confidence, which may be viewed in alignment with the core principles of social cognitive theory. Self-efficacy suggests that individuals’ beliefs in their capabilities can be progressively strengthened [38]. As our GenAI prototype was structured with phased task decomposition, for example, preloaded health templates, and provided immediate feedback mechanisms, for example, step-by-step prompt guidance, these factors likely contributed to older adults building confidence during the experience. Older adults’ belief in technological efficacy may be reinforced through gradual, experience-based accumulation when technological interactions are tailored to their cognitive capacities [39].

GenAI health videos offer a personalized visualization of health information that is often complex or difficult to access. This capability has the potential to enhance the PU of the technology, a factor that is widely understood to influence BI. Beyond mere information delivery, our findings suggest that multimodal GenAI content plays a crucial role in satisfying the emotional and cultural needs of older users. This resonance is vital for sustained engagement, as multimodal learning is most effective when it addresses the user’s underlying emotional context, a pattern also observed in how younger generations engage with multimodal platforms such as Douyin (ByteDance) for skill learning [40]. Observations suggest that while some participants initially expressed reservations about the complexity of the tools, their acceptance and willingness to use the technology appeared to increase alongside a progressive learning curve [41]. The unified theory of acceptance and use of technology, where experience acts as a critical moderating variable, posits that accumulated familiarity with technologies can substantially improve users’ attitudes [42]. Our work proposes that encouraging older adults to adopt GenAI for personal health purposes may necessitate continuous guidance and support, as well as patience, to help them cultivate confidence and practical experience. This further suggests that GenAI functions should be considered as part of a sociotechnical intervention strategy that supports older adults’ digital capabilities through both social and technical means. The multimodality of GenAI videos is also thought to contribute to technological confidence and adoption. The media richness theory by Sun and Cheng [43] proposes that high-richness media, such as animation and voice interaction, may be better suited to meet older adults’ cognitive needs. While textual data remains the dominant GenAI modality, the maturation of AI-supported interactive technologies, for example, video generation, multimodal fusion, and adaptive learning algorithms, is expected to lead to the increased application of multimodal GenAI in older adults’ health education and management.

### GenAI Videos as a Potential Virtual Companion and Access to Health Experts

As global aging intensifies, we can witness the potential of GenAI video tools as a potential virtual companion, while the prevalence of older adults living alone has risen markedly [44]. These individuals often face greater challenges in accessing health knowledge through digital technologies due to the absence of familial or societal support networks [45]. GenAI technologies may help mitigate this gap by providing algorithm-driven personalized health guidance, partially substituting traditional interpersonal support functions [46].

However, the sociotechnical implications of GenAI present a paradoxical duality. From a positive perspective, it helps address immediate gaps in health service accessibility for older adults living alone by providing basic health information and guidance when human support is unavailable. While not a complete replacement for professional care or family support, these GenAI tools can serve as a temporary solution that enables older adults to access health knowledge and manage certain aspects of their well-being independently [47]. The research by Niehaves and Plattfaut [48] suggests that digital technologies can compensate for social support deficits. Similarly, the work by Satariano and Scharlach [49] highlights how technology enhances older adults’ autonomy. The interaction between older adults and GenAI tools can be further understood through the lens of “reverse generative learning.” This approach emphasizes that intergenerational digital inclusion can be reconstructed when technology serves as a bridge, allowing older adults to develop digital readiness through creative engagement with AI [50]. Dynamic video tutorials generated by GenAI tools enable independent completion of health management tasks (such as medication management), reducing reliance on community clinics and the younger generation. On the other hand, prolonged overreliance on virtual interactions may erode real-world social connections and exacerbate emotional isolation risks [51]. Longitudinal studies reveal that while technological interventions improve functional autonomy (eg, self-management capabilities), they cannot replicate the emotional resonance of human care and potentially lead to digital isolation [52]. Recent observations indicate that some older adults living alone have reduced their frequency of visiting community health centers after adapting to GenAI, which further substantiates the contradiction [53].

To this end, technological design should adhere to the complementary principle, for example, integrating AI health videos with community volunteer service networks to provide human-based support, and at the same time, it can sustain emotional bonds with people. Cross-generational digital inclusion strategies (eg, encouraging grandchildren to participate in technology learning) could further supplement this approach, reconstructing social support networks through intergenerational interaction. These findings can underscore the necessity of embedding technological interventions within social ecosystems to avoid the pitfalls.

While older adults appreciate the value of addressing health and lifestyle issues brought by GenAI videos, they have concerns about privacy and highlight the need for human content review. These clearly reflect their uncertainty about the technology while balancing expectations for convenience against fears of misinformation or privacy violations. Finally, GenAI videos present a unique opportunity to bridge the gap between older adults and reliable health information sources. By incorporating verification mechanisms that connect AI-generated content with certified medical expertise, these tools can serve as a gateway to professional health resources. For instance, videos could include prompts encouraging users to discuss the information with their doctors or provide direct links to schedule consultations with health care providers, such as real health experts. This approach not only enhances the credibility of AI-generated content but also fosters more informed conversations between older adults and medical professionals, ultimately leading to better health outcomes.

### Ethical, Privacy, and Usability Concerns for Older Adults to Use GenAI Videos

Older adult users’ ethical concerns regarding GenAI health videos reflect profound tensions between digital-era health rights and privacy rights. Despite being aware of risks, participants still tend to accept the use of these technologies, driven by anticipated health benefits. This replicates the view of the risk perception theory by Slovic [54], wherein individuals tolerate higher risks when potential benefits are sufficiently significant. However, the “black box” nature of AI algorithms renders trust establishment that is heavily dependent on external authorities (eg, the guidance from community workers), which exposes the gap in older adults’ algorithmic interpretability rights [55]. Intergenerational differences further complicate the ethical issues. Older adults who are living alone exhibit different risk perceptions when compared to those cohabiting with family members, which prompts the necessity of the development of participatory governance frameworks [56].

Our study found that older adults’ acceptance of GenAI is influenced by privacy concerns. These concerns mainly stem from worries about personal data security, particularly regarding the storage and potential misuse of sensitive health information, which are consistent with recent findings [57]. Many older users distrust algorithmic systems, fearing accidental data leaks and questioning the reliability of AI-generated health advice. These concerns are further exacerbated by potential algorithmic biases. To address these challenges, GenAI can enhance older adults’ trust by establishing transparent data governance frameworks that clearly explain how personal information is collected, processed, and protected.

On the other hand, our findings demonstrate that older adults’ use of GenAI video tools follows a process of constructing cognitive perceptions of technological controllability, for example, the needs of adjusting playback speed and the verbal language used in narratives. The interactions between seniors and the GenAI should balance between features and aging-friendly design; for instance, step-by-step guidance, visual support, and accessible user interfaces are crucial for older adults to gain momentum in learning AI tools. In practice, the transition from passive experimentation to active cocreation can be facilitated to inform better design.

Health videos generated by GenAI may contain misleading or inaccurate information, which could disproportionately affect older adults with lower digital literacy [57]. This phenomenon is often associated with GenAI’s hallucinations, which may lead to outdated or inaccurate information. Such risks can be mitigated through the implementation of expert validation systems, where health care professionals review AI outputs. In addition, older adults should be educated to understand the limitations of AI-generated advice, and therefore, older adults’ AI literacy will be an important topic for reducing the impact of misinformation provided by GenAI.

### Limitations and Future Work

This study has several limitations. First, the urban community-focused sample may not capture access disparities of AI among rural or cross-border older adult populations, potentially limiting the generalizability of findings. Second, the short intervention period restricts the observation of long-term dynamics in technological usage. Third, reliance on self-reported data may introduce social desirability bias, possibly overestimating actual technology acceptance levels. Future work should expand to urban-rural and cross-border contexts to enhance sample diversity and use a longitudinal research design, for example, using diary methods and behavioral logs to track long-term usage patterns. The integration of multimodal data (eg, eye-tracking and physiological measurements) can be alternatives to measure the use of GenAI by this cohort.

### Conclusions

Our work makes a unique contribution to older adults’ health by examining the potential of GenAI videos in supporting older adults’ self-management and highlighting important considerations for future implementation. Currently, AI-generated videos show a promising future of empowering older adults to take an active role in managing their health despite several concerns. As AI technologies continue to advance, their thoughtful applications in aging populations can create new possibilities for accessible, personalized, and effective health education while respecting older adults’ unique needs and preferences. Future research should explore the longitudinal effects of AI-generated videos on older adults’ health outcomes, as well as the feasibility of better integrating these tools with health care systems and social support networks. It is also crucial to reduce hallucinations and misinformation in GenAI, especially for older adults with limited AI literacy. The use of GenAI videos as a learning resource for older adults in other contexts also remains a rich area of exploration for future work in older adults’ health, as do the ethicolegal implications of this kind of AI use.

## Data Availability

The author confirms that all data generated or analyzed during this study are included in this published paper. Furthermore, primary and secondary sources and data supporting the findings of this study were all publicly available at the time of submission.

## Appendix

Multimedia Appendix 1 GenAI health video research questionnaire. GenAI: generative artificial intelligence.

## Abbreviations

AI: artificial intelligence
BI: behavioral intention
GenAI: generative artificial intelligence
PEOU: perceived ease of use
PU: perceived usefulness
RTA: reflexive thematic analysis
