# Supplementary material for: Evaluating the Use of Generative AI Videos for Health Self-Management of Older Adults: Mixed Methods Study
Source: JMIR Aging. 2026 Mar 4;9:e88005. doi: 10.2196/88005 (PMC13000384; doi:10.2196/88005)
Supplement: Multimedia Appendix 1 [file aging_v9i1e88005_app1.docx]

### GenAI health video research questionnaire

## Pretest questionnaire (pre-intervention)

Dear participants:

Hello! We hope to understand your experience of interacting with GenAI health videos through this questionnaire, especially your attitude and confidence in the technology of GenAI-generated health videos. Please select the number that best matches your situation on the right according to your true thoughts (1=strongly disagree, 2=disagree, 3=neutral, 4=agree, 5=strongly agree). There are no right or wrong answers to all responses. Thank you for your participation!

### Part 1: Basic information

1. Your age: _________ years old
2. Your gender: □male □female □other
3. What is your current physical condition? □ healthy □with mild illnesses □with chronic diseases □other________
4. What is your current living arrangement? □ living alone □living with family □nursing home □other________
5. What is your educational background? □ primary school □junior high school □senior high school □college/vocational college □university and above
6. Have you ever used AI tools? □yes → if yes, what do you usually use them for? (multiple choices allowed) □calls □social media □information searching □entertainment □other________□ no

### Part 2: Technology acceptance model (TAM)

#### Perceived usefulness (PU)

| **Items** | **strongly disagree** | **disagree** | **neutral** | **agree** | **strongly agree** |
| --- | --- | --- | --- | --- | --- |
| 1. GenAI health videos can help me understand health knowledge and manage my health. | 1 | 2 | 3 | 4 | 5 |
| 2. GenAI health videos are more effective in understanding health knowledge than traditional methods (e.g., books, lectures). | 1 | 2 | 3 | 4 | 5 |
| 3. The health information provided by GenAI health videos is useful to me. | 1 | 2 | 3 | 4 | 5 |

#### Perceived ease of use (PEOU)

| **Items** | **strongly disagree** | **disagree** | **neutral** | **agree** | **strongly agree** |
| --- | --- | --- | --- | --- | --- |
| 4. The production operation and content of GenAI health videos are simple and easy to understand. | 1 | 2 | 3 | 4 | 5 |
| 5. I can easily understand the process of using GenAI health videos. | 1 | 2 | 3 | 4 | 5 |
| 6. I think learning to generate health videos with GenAI does not require too much time and effort. | 1 | 2 | 3 | 4 | 5 |

#### Behavioral intention (BI)

| **Items** | **strongly disagree** | **disagree** | **neutral** | **agree** | **strongly agree** |
| --- | --- | --- | --- | --- | --- |
| 7. I am willing to use GenAI health videos to learn health knowledge. | 1 | 2 | 3 | 4 | 5 |
| 8. I am willing to recommend GenAI and GenAI health videos to family or friends. | 1 | 2 | 3 | 4 | 5 |

### Part 3: Self-efficacy

#### Technological confidence and learning ability

| **Items** | **strongly disagree** | **disagree** | **neutral** | **agree** | **strongly agree** |
| --- | --- | --- | --- | --- | --- |
| 9. I believe I can try to learn to use GenAI to generate health videos. | 1 | 2 | 3 | 4 | 5 |
| 10. I believe I can understand the content of health videos generated by GenAI. | 1 | 2 | 3 | 4 | 5 |
| 11. If someone teaches me to use GenAI tools, I believe I can try to complete the relevant operations. | 1 | 2 | 3 | 4 | 5 |
| 12. I am confident in obtaining the health video content I need with GenAI. | 1 | 2 | 3 | 4 | 5 |

#### Task mastery

| **Items** | **strongly disagree** | **disagree** | **neutral** | **agree** | **strongly agree** |
| --- | --- | --- | --- | --- | --- |
| 13. I believe I can clearly express my needs and make GenAI generate health videos that suit my needs. | 1 | 2 | 3 | 4 | 5 |
| 14. I think I have the ability to participate in the creation of GenAI health videos. | 1 | 2 | 3 | 4 | 5 |

### Part 4: Impact on health management learning

| **Items** | **strongly disagree** | **disagree** | **neutral** | **agree** | **strongly agree** |
| --- | --- | --- | --- | --- | --- |
| 15. I take the initiative to learn health knowledge in daily life. | 1 | 2 | 3 | 4 | 5 |
| 16. I can obtain the health information I need through GenAI tools in the future. | 1 | 2 | 3 | 4 | 5 |
| 17. I think GenAI tools can help me obtain health knowledge more easily. | 1 | 2 | 3 | 4 | 5 |

## Posttest questionnaire (post-intervention)

After this iterative GenAI design workshop, w**e** want to understand your experience of interacting with GenAI health videos. Please select the number that best matches your situation on the right according to your real experience (1=strongly disagree, 2=disagree, 3=neutral, 4=agree, 5=strongly agree). Thank you for your feedback!

### Part 1: Technology acceptance model (TAM)

#### Perceived usefulness (PU)

| **Items** | **strongly disagree** | **disagree** | **neutral** | **agree** | **strongly agree** |
| --- | --- | --- | --- | --- | --- |
| 1. After participating in the workshop, GenAI health videos help me understand and manage health problems. | 1 | 2 | 3 | 4 | 5 |
| 2. GenAI health videos are more effective than traditional methods (e.g., books, lectures). | 1 | 2 | 3 | 4 | 5 |
| 3. The health information provided by GenAI health videos is useful to me. | 1 | 2 | 3 | 4 | 5 |

#### Perceived ease of use (PEOU)

| **Items** | **strongly disagree** | **disagree** | **neutral** | **agree** | **strongly agree** |
| --- | --- | --- | --- | --- | --- |
| 4. The production operation and content of GenAI health videos are simple and easy to understand. | 1 | 2 | 3 | 4 | 5 |
| 5. I have understood the process of generating health videos with GenAI. | 1 | 2 | 3 | 4 | 5 |
| 6. Now I think learning to generate health videos with GenAI does not require too much time and effort. | 1 | 2 | 3 | 4 | 5 |

#### Behavioral intention (BI)

| **Items** | **strongly disagree** | **disagree** | **neutral** | **agree** | **strongly agree** |
| --- | --- | --- | --- | --- | --- |
| 7. Now I am more willing to try using GenAI videos to learn health knowledge. | 1 | 2 | 3 | 4 | 5 |
| 8. I am willing to recommend GenAI and GenAI health videos to family or friends. | 1 | 2 | 3 | 4 | 5 |

### Part 2: Self-efficacy

#### Technological confidence and learning ability

| **Items** | **strongly disagree** | **disagree** | **neutral** | **agree** | **strongly agree** |
| --- | --- | --- | --- | --- | --- |
| 9. After participating in the workshop, I believe I can understand the process of generating health videos with GenAI. | 1 | 2 | 3 | 4 | 5 |
| 10. I believe I can understand the content of health videos generated by GenAI. | 1 | 2 | 3 | 4 | 5 |
| 11. If someone teaches me to use GenAI tools, I believe I can try to complete the relevant operations. | 1 | 2 | 3 | 4 | 5 |
| 12. I am confident in obtaining the health video content I need through GenAI tools. | 1 | 2 | 3 | 4 | 5 |

#### Task mastery

| **Items** | **strongly disagree** | **disagree** | **neutral** | **agree** | **strongly agree** |
| --- | --- | --- | --- | --- | --- |
| 13. I believe I can clearly express my needs and make GenAI generate health videos that suit my needs. | 1 | 2 | 3 | 4 | 5 |
| 14. I think I can participate in the creation of GenAI health videos. | 1 | 2 | 3 | 4 | 5 |

### Part 3: Impact on health management learning

| **Items** | **strongly disagree** | **disagree** | **neutral** | **agree** | **strongly agree** |
| --- | --- | --- | --- | --- | --- |
| 15. After participating in the workshop, I am more willing to take the initiative to learn health knowledge. | 1 | 2 | 3 | 4 | 5 |
| 16. I can obtain the health information I need through GenAI videos in the future. | 1 | 2 | 3 | 4 | 5 |
| 17. GenAI videos can help me obtain health knowledge more efficiently. | 1 | 2 | 3 | 4 | 5 |
| 18. After participating in the workshop, I feel that my ability to use GenAI and manage health has improved. | 1 | 2 | 3 | 4 | 5 |
| 19. After participating in the workshop, I think GenAI health videos are helpful for my health management. | 1 | 2 | 3 | 4 | 5 |

### Part 4: Iterative design feedback

| **Items** | **strongly disagree** | **disagree** | **neutral** | **agree** | **strongly agree** |
| --- | --- | --- | --- | --- | --- |
| 20. I am satisfied with the final health videos generated by GenAI. | 1 | 2 | 3 | 4 | 5 |
| 21. I am satisfied with this workshop activity. | 1 | 2 | 3 | 4 | 5 |
| 22. Multiple revisions can achieve the effect I want. | 1 | 2 | 3 | 4 | 5 |
| 23. I am willing to participate in more similar workshops. Reasons: | 1 | 2 | 3 | 4 | 5 |
| 24. The health videos generated by GenAI make me feel friendly and approachable. | 1 | 2 | 3 | 4 | 5 |
